# Use of Electrodiagnostics in the Diagnosis and Follow-Up of Brachial Plexus Syndrome in a Calf

**DOI:** 10.3390/vetsci9030136

**Published:** 2022-03-15

**Authors:** Marilena Bolcato, Mariana Roccaro, Joana G. P. Jacinto, Angelo Peli, Arcangelo Gentile, Ezio Bianchi

**Affiliations:** 1 Department of Veterinary Medical Sciences, Alma Mater Studiorum University of Bologna, 40064 Bologna, Italy; joana.goncalves2@studio.unibo.it (J.G.P.J.); arcangelo.gentile@unibo.it (A.G.); 2 Department for Life Quality Studies, Alma Mater Studiorum University of Bologna, 47921 Rimini, Italy; mariana.roccaro2@unibo.it (M.R.); angelo.peli@unibo.it (A.P.); 3 Department of Veterinary Medical Sciences, University of Parma, 43100 Parma, Italy; ezio.bianchi@unipr.it

**Keywords:** bovine, neurology, brachial plexus, traumatic injury, electromyography, motor nerve conduction study

## Abstract

Electrodiagnostic testing by using electromyography (EMG) and nerve conduction studies (NCS) is essential in the evaluation of patients with traumatic brachial plexus injury as it facilitates the localization of the lesion and the prognosis. In this case report, we present a long-term electrodiagnostic follow-up of a 5-day-old female Holstein calf with brachial plexus syndrome. Electrodiagnostic studies were carried out at 2 weeks, 5 weeks, 7 months and 12 months after admission. Initially, EMG confirmed the damage to the brachial plexus, potentially indicating a condition of neurotmesis or axonotmesis. However, motor NCS and the repeated electrodiagnostic follow-up, along with the evolution of the clinical signs, allowed a more favorable diagnosis of axonotmesis to be made. In fact, EMG showed a slow but gradual reduction and finally the disappearance of spontaneous pathological activity, while motor NCS revealed an increase in the amplitude and areas of the compound muscle action potentials. The animal was deemed fully recovered 12 months after admission. To the authors’ knowledge, this is the first report on the use of motor NCS in bovine medicine and it demonstrates that electrodiagnostics represent a useful and practical tool for the evaluation and prognosis of brachial plexus injury cases in cattle.

## 1. Introduction

The brachial plexus is a complex network of nerves responsible for the innervation of the thoracic limbs. It is located medial to the shoulder and cranial to the first rib [1].

In cattle, the main nerves constituting the brachial plexus originate from the 6th to the 8th cervical spinal cord segments (C6–C8) and the 1st thoracic spinal cord segment (T1), whereas C5 and T2 segments make minor contributions [2]. Both sensory and motor nerves originate from the brachial plexus that innervate the thoracic limb and part of the trunk. In addition, the sympathetic fibers that innervate the eye, originating from lower motor neurons located in the first three thoracic segments, travel along the roots of the brachial plexus as they exit the vertebral canal [1].

Total brachial plexopathy leads to paralysis and analgesia of the limb, Horner’s syndrome, and loss of the cutaneous trunci reflex on the same side [3]. Otherwise, the clinical signs associated with partial impairment of the plexus depend upon the specific nerves injured [4].

In particular, avulsion of the ventral roots of T1 or of the T1 spinal nerve causes miosis due to the interruption of the GVE (General Visceral Efferent) preganglionic neuronal axons located there, which provide sympathetic innervation to the eye. Simultaneous damage of the GVE neurons to the head and GSE (General Somatic Efferent) neurons to the thoracic limb can therefore only be observed in ventral root lesions. However, the same GSE-LMN (Lower Motor Neuron) paralysis observed in the thoracic limb would occur if the injury were located within the brachial plexus, but this lesion could not have affected the GVE innervation of the head because those preganglionic axons leave the GSE axons just distal to the intervertebral foramen, where the ramus communicans leaves the spinal nerve to join the cranial portion of the thoracic sympathetic trunk [5].

The loss of the cutaneous trunci reflex ipsilateral to the lesion occurs with injury of the C8 and T1 ventral spinal nerve roots, which causes the interruption of the lateral thoracic nerve innervation of the cutaneous trunci muscle [1].

In bovine medicine, the brachial plexus syndrome (BPS) occurs mostly as a consequence of excessive traction at the time of calving [4] or other traumatic injuries [6,7], but it can also be caused by prolonged recumbency [3], intramuscular injection [8], iatrogenic local anesthesia [9], neoplasia [3,4,5,6], and infections [10].

BPS is characterized by unilateral thoracic limb paresis, with muscle atrophy beginning in about one week. The motor syndrome is accompanied by sensory dysfunction, whose distribution in the affected limb is very useful for localizing the most damaged part [1].

Electromyography (EMG) and nerve conduction studies (NCS) are reported as the most sensitive tests for the evaluation of axonal damage, enabling a more precise localization of the lesion and follow-up, not only in human medicine but also in veterinary medicine [11]. In human medicine, EMG and NCS provide the best diagnostic and prognostic results if performed no sooner than 3–4 weeks after injury onset. On EMG, the presence of voluntary motor unit potentials leads to a better prognosis than the total absence of motor unit action potentials (MUAPs) [12]. In veterinary medicine, the evaluation of voluntary muscle contraction through MUAPs is not applicable due to poor patient compliance. However, EMG is still useful for the evaluation of spontaneous activity [13]. On NCS, in traumatic brachial plexus injuries, the amplitude of compound muscle action potentials (CMAPs) is generally low, and it is related to the total amount of functional muscle fibers. Serial testing in conjunction with repeated physical examination every few months allows to monitor and quantify ongoing reinnervation or denervation [12].

Despite its usefulness, the use of electrodiagnostic testing is still very limited in bovine medicine and, to the authors’ knowledge, motor NCS have never been reported in bovine clinical practice so far. The only study performed on calves provides only motor nerve conduction velocity (mNCV) parameters for the radial and the sciatic/common peroneal nerve in healthy subjects [14].

In this case report, we present the clinical and electrodiagnostic findings and follow-up of a BPS consequent to brachial plexus injury in a calf. The use of motor NCS, for the first time in bovine medicine, is presented. With this report, we aim to advocate for the usefulness and feasibility of electrodiagnostic studies in the evaluation and prediction of the clinical course in bovine medicine.

## 2. Materials and Methods

### 2.1. Case Description

A 5-day-old female Holstein calf was referred and admitted to the teaching hospital of the Department of Veterinary Medical Sciences of the University of Bologna due to acute and severe lameness of the left thoracic limb. The owner reported that the day before admission the calf had been trapped with its left thoracic limb in the feeding trough of its individual pen and was lame after being freed. Immediately afterwards, the calf had received an anti-inflammatory injection, but seeing no improvement, the owner decided to have the animal hospitalized. No information was provided about the delivery.

**Figure 1:**
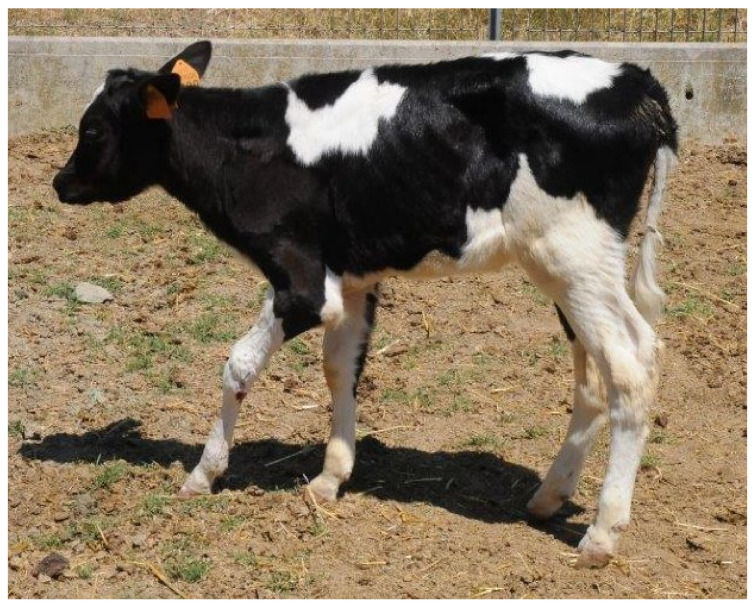
Presentation of the calf at admission. Observe the posture of the left thoracic limb: flexed and dropped elbow, flexed carpus, overextended fetlock joint.

At clinical examination, the animal was in good general condition and had normal demeanor. The prescapular lymph nodes were bilaterally and moderately increased in size, but not painful or warm. The body temperature, pulse and respiratory rate were normal. The left thoracic limb posture was peculiar: the elbow was flexed and dropped, the carpus was flexed, and the fetlock joint was overextended (Figure 1). Palpation and manipulation of the entire limb did not elicit crepitation or pain; all joints appeared clinically normal and could be flexed and extended passively without difficulty.

At neurological examination, moderate atrophy of the shoulder musculature and of the triceps was detected. The affected limb was severely paretic, and the contact of the hoof with the ground was very limited when walking. Miosis was observed in the left eye only (Horner’s syndrome). The flexor and extensor carpi radialis reflexes were reduced and postural reactions were absent in the affected limb. An area of analgesia was detected over the dorsum of the metacarpus and phalanges. The cutaneous trunci reflex was absent on the left side.

The clinical picture was consistent with a lesion of the left brachial plexus. In view of the information provided by the owner, a traumatic avulsion of the roots of the nerves that form the brachial plexus was suspected as a consequence of the repeated movements of traction and/or abduction of the limb, made by the animal while being trapped, in an attempt to free itself.

Given the calf’s good general condition, its ability to stand and move autonomously, and the owner’s request to do everything possible for the animal, it was decided to proceed with medical treatment.

A vitamin-based treatment was implemented: vitamin E and selenium (10 mL/week for 5 weeks, Selevit, Fatro S.p.A., Ozzano dell’Emilia, Italy), vitamins A, D, E (5 mL/week for 2 weeks, Adisole, Ceva Salute Animale S.p.A., Agrate Brianza, Italy) and vitamin B (5 mL/week for 2 weeks, Dodicile, Fatro S.p.A., Ozzano dell’Emilia, Italy) were administered subcutaneously to support nerve healing [8,15].

In addition, a splint bandage that extended from the hoof to the elbow was applied on the injured limb. Due to the development of pressure sores on the elbow after 15 days, the splint was replaced by a simple bandage. The latter stayed in place for six months, but it was removed for 1 h twice a week, when physiotherapy, consisting in the extension and flexion of all joints as well as the massaging of all muscles for 15 min, was practiced.

### 2.2. Electrodiagnostic Investigation

Electrodiagnostic investigations (Myoquick, Micromed S.p.A., Mogliano Veneto, Italy), were performed 2 weeks (EMG), 5 weeks (motor NCS), 7 months (EMG and motor NCS) and 12 months (EMG and motor NCS) after admission. EMG did not require any sedation, whereas for the motor NCS the calf was sedated with xylazine (0.5 mg/100 Kg, i.m., Rompum 2%, Bayer AG, Leverkusen, Germany).

EMG was performed to evaluate the spontaneous activity of *biceps brachii*, *triceps brachii*, *flexor carpi ulnaris* and *flexor carpi radialis* muscles of the left thoracic limb, which are innervated by the brachial plexus originating musculocutaneous, radial, ulnar and median nerves, respectively.

**Figure 2:**
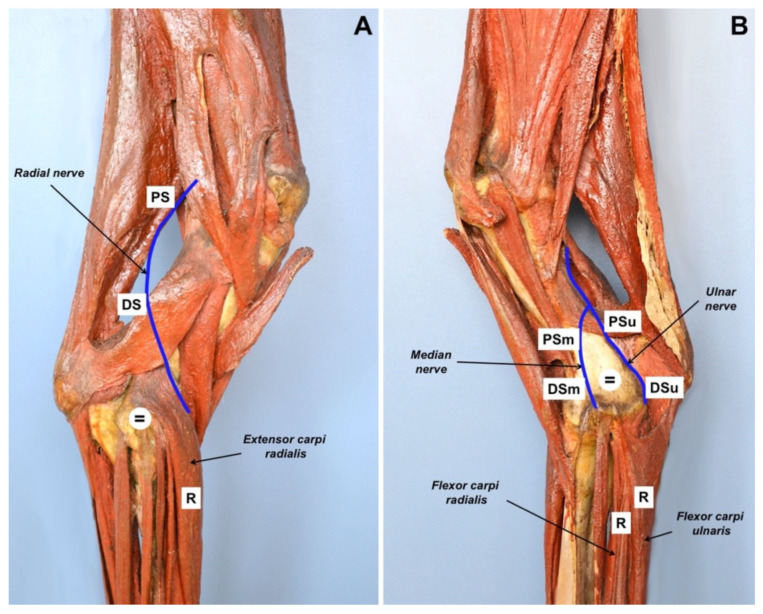
Photographs demonstrating the position of electrodes during motor NCS on an anatomical model of bovine right thoracic limb. (**A**) Lateral view. The stimulating needles for the radial nerve were placed at the angle between the head of the humerus and the ventral portion of the scapula (PS) and at the third distal of the humerus, caudally to the brachialis muscle (DS). The position of ground needle (=) and recording needle (R) is also shown. (**B**) Medial view. The stimulating needles for the ulnar nerve (PSu, DSu) were placed caudally to the medial humerus epicondyle; the stimulating needles for the median nerve (PSm, DSm) were placed cranially to the medial humerus epicondyle. The position of ground needle (=) and recording needles (R) is also shown. (Courtesy of Domestic Animal Anatomy Collection, Sistema Museale d’Ateneo, *Alma Mater Studiorum*, University of Bologna).

Motor NCS (CMAPs) were recorded from the *extensor carpi radialis* [14], *flexor carpi ulnaris* and *flexor carpi radialis* muscles, after stimulation of the radial, ulnar and median nerves, respectively. In particular, the radial nerve was stimulated proximally at the angle between the head of the humerus and the ventral portion of the scapula and at the distal third of the humerus, caudally to the *brachialis* muscle; the ulnar nerve was stimulated caudally to the medial humerus epicondyle; the median nerve was stimulated cranially to the medial humerus epicondyle (Figure 2). Stimulating, recording and ground electrodes were 25 mm long monopolar needles. Recording needles were placed using a belly tendon montage. The ground needle was always placed between the stimulating and the recording needles. Nerves were stimulated with supramaximal rectangular pulses of 0.1 ms duration. The lower and upper cut-off frequencies applied to the recording were 0.1 Hz and 5 kHz, respectively. Peak-to-peak CMAP amplitudes were measured; CMAP areas were calculated as the areas delimited by the deflections (negative and positive) and the baseline. Motor NCS were carried out on both thoracic limbs, with the right limb being used as healthy control.

## 3. Results

**Figure 3:**
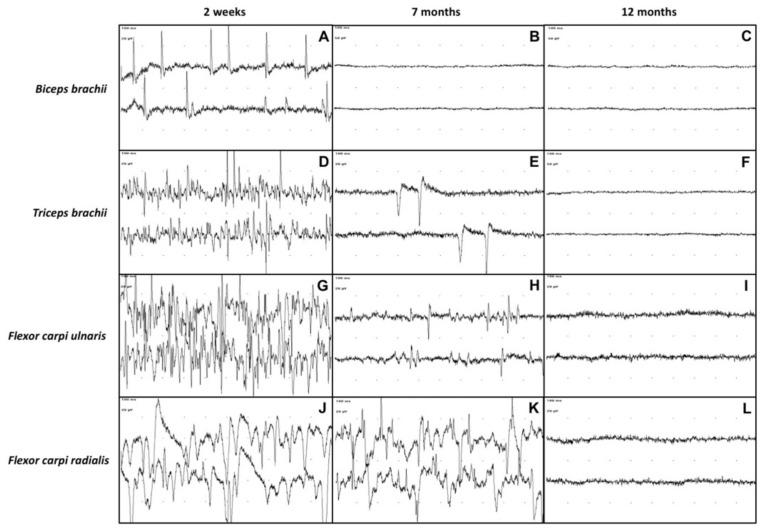
EMG recording of the left thoracic limb’s muscles (*biceps brachii, triceps brachii, flexor carpi ulnaris, flexor carpi radialis*) at 2 weeks, 7 months and 12 months after admission. Sweep speed: 10 ms/div. *Biceps brachii* muscle at 2 weeks (**A**), 7 months (**B**) and 12 months (**C**) after admission. *Triceps brachii* muscle at 2 weeks (**D**), 7 months (**E**) and 12 months (**F**) after admission. Third row: *Flexor carpi ulnaris* muscle at 2 weeks (**G**), 7 months (**H**) and 12 months (**I**) after admission. *Flexor carpi radialis* muscle at 2 weeks (**J**), 7 months (**K**) and 12 months (**L**) after admission. An evident reduction of spontaneous pathologic activity can be observed at 7 months after admission up to normalization of EMG tracings in all the recorded muscles at 12 months after admission.

At the first electrodiagnostic investigation, performed 2 weeks after admission, EMG revealed the presence of moderate to severe spontaneous pathologic activity in all the evaluated muscles of the left thoracic limb (Figure 3A,D,G,J).

**Figure 4:**
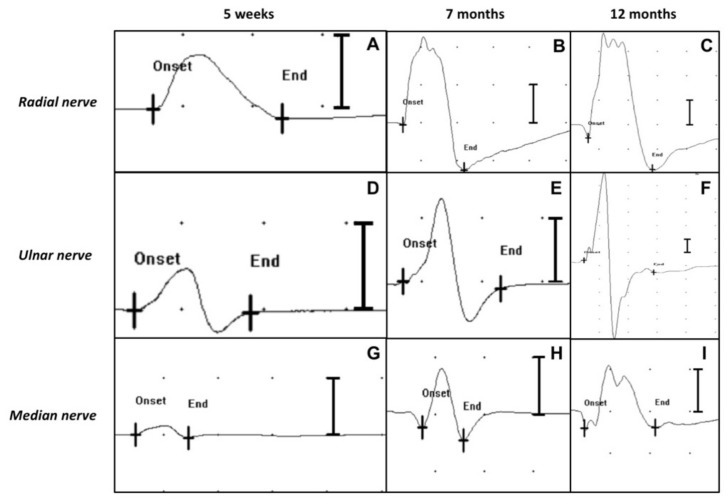
Motor NCS (CMAPs) of the left thoracic limb’s nerves (radial nerve, ulnar nerve, median nerve) at 5 weeks, 7 months and 12 months after admission (distal stimulation). Sweep speed: 5 ms/div. Sensitivity: 2 mV/div. A progressive increase of the amplitudes and areas of the CMAPs over time can be observed in all the tested nerves. (**A**) CMAP amplitude 1.8 mV, area 6.3 uVs; (**B**) CMAP amplitude 7.1 mV, area 23.1 uVs; (**C**) CMAP amplitude 11.2 mV, area 45.7 uVs; (**D**) CMAP amplitude 1.5 mV, area 3 uVs; (**E**) CMAP amplitude 3.9 mV, area 7.5 uVs; (**F**) CMAP amplitude 25.1 mV, area 54.3 uVs; (**G**) CMAP amplitude 0.5 mV, area 0.9 uVs; (**H**) CMAP amplitude 2.5 mV, area 5.3 uVs; (**I**) CMAP amplitude 3.1 mV, area 8.9 uVs.

**Figure 5:**
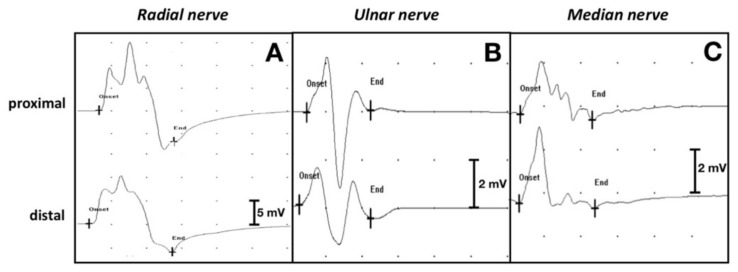
Motor NCS (CMAPs) of the right thoracic limb’s nerves (healthy control) at 5 weeks after admission. Sweep speed: 5 ms/div. CMAP amplitudes, areas, and nerve conduction velocity (NCV) for each tested nerve are reported: (**A**) Radial nerve (proximal CMAP amplitude 23.7 mV, area 73.3 uVs; distal CMAP amplitude 16.3 mV, area 62.4 uVs; NCV 53.6 m/s). Sensitivity: 5 mV/div; (**B**) Ulnar nerve (proximal CMAP amplitude 5.5 mV, area 9.3 uVs; distal CMAP amplitude 3.2 mV, area 7.8 uVs; NCV 44.4 m/s). Sensitivity: 2 mV/div; (**C**) Median nerve (proximal CMAP amplitude 2.8 mV, area 6.6 uVs; distal CMAP amplitude 3.9 mV, area 8 uVs; NCV 125 m/s). Sensitivity: 2 mV/div.

The motor NCS performed 5 weeks after admission showed that the left thoracic limb CMAPs of the radial, ulnar and median nerves were significantly reduced in amplitude and area (Figure 4A,D,G), compared with the right thoracic limb (Figure 5A–C).

Clinical improvement became evident only after three months of observation: the limb started to show a voluntary weak movement of the elbow joint and the atrophy of the triceps, supraspinatus and infraspinatus muscles gradually decreased. Moreover, the calf regained nociceptive sensation over the dorsum of the metacarpus and phalanges.

At 7 months after admission, the calf was able to stand on four legs, although the gait was still quite unsteady. The flexor and extensor carpi radialis reflexes of the affected limb, as well as the postural reactions, were still slightly reduced. The sensory testing detected nociception on the entire limb and cutaneous trunci reflex was present bilaterally. The triceps were increased in volume. The cutaneous trunci reflex was bilaterally present and no miosis was observed in the left eye. On EMG, the only muscles showing spontaneous pathologic activity at the EMG were the *triceps brachii*, *flexor carpi ulnaris* and *flexor carpi radialis* (Figure 3E,H,K). Moreover, the pathologic activity was less intense than in the previous observation. Interestingly, the EMG activity of the *biceps brachii* was completely normal (Figure 3B). The motor NCS of the left thoracic limb showed an increase in the CMAPs amplitude and area (Figure 4B,E,H), compared with the previous NCS. 

Twelve months after admission, the EMG detected no spontaneous pathologic activity in the affected limb (Figure 3C,F,I,L). The CMAPs amplitude and area of the left thoracic limb’s nerves had further increased compared to the previous recordings and were similar to those of the healthy right thoracic limb (Figure 4C,F,I). At clinical examination, the animal exhibited completely normal posture and motor functions. After a thorough neurological investigation, the animal was deemed clinically recovered.

## 4. Discussion

EMG and NCS are recognized ancillary diagnostic tests for determining the localization and severity of nerve injuries [16], as well as for monitoring the effectiveness of treatment [10]. However, due to the need for specific devices and specialized expertise, these techniques have so far not become routine tools in bovine clinical practice.

The present study describes a long term electrodiagnostic follow-up (1 year) in a calf affected by traumatic brachial plexus syndrome, which allowed to determine the injured nerves, to estimate the extent of the lesion and to monitor nerve regeneration. 

At first examination, the clinical findings were not in agreement with the history reported by the owner. Indeed, the atrophy observed in the shoulder muscles and in the triceps appeared too severe for an injury sustained only the day before. In light of this finding, the authors suspected that the brachial plexus injury might have occurred earlier, possibly at the calf’s birth or even during intrauterine life. In human medicine, BPS in newborns is reported worldwide in 0.1 to 8.1 per 1000 live births and the mechanisms of injury include maternal, obstetric, and infant factors that apply traction on the plexus [17]. Interestingly, several studies have implied that intrauterine maladaptation (e.g., fetal macrosomia) may also play a role in brachial plexus injury in the newborn [18,19].

In the first two-three months of hospitalization, the clinical findings were strongly suggestive of a poor prognosis, with the involvement of large segments of the affected nerves. In particular: (i) the dropped elbow and the atrophy of the *triceps brachii* muscle were consistent with the impairment of the radial nerve (corresponding to spinal tract C7-C8–T1); (ii) the dropped elbow was consistent with the impairment of the musculocutaneous nerve (C6–C7); (iii) the hyperextension of carpus, fetlock and digits was consistent with the impairment of the median and ulnar nerves (C7–C8–T1 and C8–T1–T2, respectively); (iv) the reduction of the limb reflexes (both the flexor and the extensor carpi radialis reflex), associated with analgesia detected over the dorsum of metacarpus and phalanges, was consistent with the impairment of a group of nerves including the radial (C7–C8–T1), median and ulnar (C7–C8–T1–T2) and axillary (C6–C7) nerves. All the aforementioned nerves contribute to the complex network that constitutes the brachial plexus [2]. The partial Horner’s syndrome (T1) and the loss of the cutaneous trunci reflex (C8–T1), ipsilateral to the lesion, were additional consequences of the involvement of the caudal portion of the brachial plexus.

Electrodiagnostics was used to determine the severity of the lesion and to monitor the course of the disease. The spontaneous pathologic activity at the EMG in all the evaluated muscles of the left front limb, observed 2 weeks after admission, confirmed the presence of nerve damage and the extent of the trauma, potentially indicating a condition of neurotmesis or axonotmesis.

Among nerve injuries, neurotmesis is the one with the worst prognosis: both the axon and the connective tissue are damaged and nerve regeneration is unlikely because of the destruction of the basal membrane [8]. Moreover, the brachial plexus originates from cervical and upper thoracic nerve roots, and these respond in a different way to traumatic lesions compared to peripheral nerves. In fact, nerve root avulsion, resulting in axon and myelin sheath rupture of both dorsal and ventral roots at the junction with the spinal cord, determines a condition of neurotmesis and recovery is only possible following surgery. On the other hand, proximal axonal lesions determine distal axonal degeneration, with changes similar to peripheral nerve injuries [20].

Axonotmesis is the type of nerve injury where the axon is separated from the neuronal cell body, resulting in the degeneration of its distal end (Wallerian degeneration) and loss of conduction capacity in 3 to 5 days. Nevertheless, the nerve connective tissue elements (epineurium and, sometimes, perineurium and endoneurium) are unaffected and axonal regeneration is possible [1].

To make a more accurate diagnosis and prognosis, a motor NCS was performed 5 weeks after admission, and low amplitude CMAPs were elicited. According to CMAP amplitude, lesions can be classified as minor axonotmesis with normal/subnormal CMAP amplitude, severe axonotmesis with severely decreased CMAP amplitude, or neurotmesis with no elicited CMAP [20]. On the basis of these findings, a diagnosis of axonotmesis was made. Given the absence in the literature of physiological and pathological reference values for CMAPs in calves, the CMAP amplitudes and areas recorded from the healthy thoracic limb were used for comparison; hence, a severe axonotmesis was hypothesized. Since the owner requested to do everything possible for the animal, it was decided to continue the treatment.

In neuromuscular damage, good nursing care plays a key role: clean and dry bedding, confinement and secure footing are recommended. Physiotherapy, passive manipulation, and frequent lifting to encourage weight-bearing are indicated to prevent muscle contractures and to stimulate blood flow to the flaccid musculature. Limb support is essential to minimize self-trauma. Moreover, a high splint or bandage may stabilize the carpus and the fetlock and facilitate weight bearing [8].

In cattle, recovery of muscular function is considered possible up to 6 months after injury [15]. In our case, the calf’s clinical conditions began to improve slowly but steadily from the third month after admission. At 7 months after admission, the clinical situation had further improved: the paresis of the left front limb was less severe compared with the previous evaluations and the analgesia of the limb had disappeared. The neurological improvement was confirmed by electrodiagnostic testing: the reduction of the spontaneous pathologic activity at the EMG and the increased amplitude and areas of the CMAPs at motor NCS in all the evaluated nerves indicated a gradual process of muscular reinnervation.

Axonal regeneration usually begins in about 1 week with a progression rate of approximately 1 to 4 mm per day [1]. Knowing this, it is possible to estimate how long it will take for signs of regeneration to become evident by measuring the distance between the site of injury and the midpoint of the denervated muscle. Assuming the slowest rate (1 mm/day), the distance in millimeters corresponds to the number of days required for regeneration [5]. In this case, the normal EMG aspect of the *biceps brachii* muscle at 7 months after admission was therefore consistent with it being the closest to the brachial plexus.

At 12 months after admission, the absence of clinical signs was accompanied by a normalization of the electrodiagnostic parameters. The absence of spontaneous pathologic activities in all tested muscle at EMG and the normalization of the amplitude and areas of the CMAPs at motor NCS indicated complete regeneration of axons and reinnervation of muscles. The patient could finally be discharged.

In this study, the motor NCS was limited to the evaluation of CMAPs because it was not possible to accurately measure motor nerve conduction velocity (NCV). In fact, in order to obtain a reliable measure, the distance between proximal and distal stimulation should be at least 10 cm [13]. Due to the anatomical constraints of young calves, it is very difficult to achieve such a distance. This problem, already reported by Schenk et al. for radial nerve [14], is even more pronounced for ulnar and median nerves, which lie in the medial side of the antebrachium and therefore can be reached only from the distal humerus/elbow for electrical stimulation. Nevertheless, an attempt to measure NCV in the healthy thoracic limb was made, given the absence in the literature of data concerning these nerves in cattle. In this case, the distance between proximal and distal stimulation sites was 7.5 cm for the radial nerve, 5 cm for the ulnar nerve and 2.5 cm for the median nerve. In light of these limitations, the reported NCV measurements should be taken with caution.

Consequently, in the affected limb, the evaluation was limited to those measurements based on single point stimulation, such as the CMAPs. Moreover, considering the type of lesion, NCV was not deemed to be such a relevant parameter. Indeed, when nerve damage is focal and not complete, the presence of some intact, fast-conducting fibers may produce only mild to moderate NCV reduction. On the contrary, CMAP amplitude and area are more sensitive indicators of the severity of nerve damage because they indirectly reflect the number of functioning nerve fibers.

## 5. Conclusions

In conclusion, in calves, brachial plexus injury can be associated with a good prognosis, even when initial neurologic and electrodiagnostic findings indicate a severe nerve root injury. Therefore, correct diagnosis and follow-up are paramount to safeguard the welfare of the animal and to prevent unnecessary culling. This is also beneficial for the farmer as calves can have a considerable economic value.

Serial clinical and possibly electrodiagnostic evaluations should be performed in the months following the trauma to detect signs of regeneration of the affected nerves. Considering the nerve length of these subjects, clinicians should allow the calf enough time for the potential functional recovery of the limb. Indeed, muscle reinnervation can take 6 to 12 months.

## Data Availability

All study data are presented in the article.
